# Green Synthesis of Silver Nanoparticles (CM-AgNPs) from the Root of *Chuanminshen* for Improving the Cytotoxicity Effect in Cancer Cells with Antibacterial and Antioxidant Activities

**DOI:** 10.3390/molecules29235682

**Published:** 2024-11-30

**Authors:** Dandan Wang, Haijing Ke, Hongtao Wang, Jingyu Shen, Yan Jin, Bo Lu, Bingju Wang, Shuang Li, Yao Li, Wan Taek Im, Muhammad Zubair Siddiqi, Haibo Zhu

**Affiliations:** 1 College of Life Sciences, Yantai University, Yantai 264005, China; whdandan@163.com (D.W.); jyshen119@163.com (J.S.); lb17861136752@163.com (B.L.); l15253511514@163.com (S.L.);; 2 School of Life Science, Nantong University, Nantong 226019, China; jinyan224@hotmail.com; 3 Department of Biotechnology, Hankyong National University, 327 Jungang-ro, Anseong-si 17579, Gyeonggi-do, Republic of Korea; wandra@hknu.ac.kr; 4 School of Public Health and Management, Binzhou Medical University, Yantai 264005, China

**Keywords:** *Chuanminshen violaceum Sheh et Shan*, green synthesis, silver nanoparticles, anti-oxidant activity, bacteriostasis, anti-cancer properties

## Abstract

The unique properties of silver nanoparticles (AgNPs), such as size, surface charge, and the ability to release silver ions, contribute to DNA damage, inducing of oxidative stress, and apoptosis in cancer cells. Thus, the potential application of AgNPs in the field of biomedicine, and cancer therapy are rapidly increasing day by day. Therefore, in this study, AgNPs were synthesized by extract of *Chuanminshen violaceum*, and then the synthesized CM-AgNPs were fully characterized. The biological activity of CM-AgNPs was investigated for antibacterial, antioxidant, and anticancer activities. The cytotoxic activity of CM-AgNPs was tested for various kinds of cancer cells including MKN45 gastric cancer cells, HCT116 human colon cancer cells, A549 human lung cancer cells, and HepG2 liver cancer cells. Among these cancer cells, the induced apoptosis activity of CM-AgNPs on HCT116 cancer cells was better and was used for further investigation. Besides, the CM-AgNPs exhibited great antioxidant activity against 1,1-diphenyl-2-picrylhydrazyl (DPPH) with 50% free radical scavenging activity, and CM-AgNPs also showed a significant antibacterial activity against *Escherichia coli* and *Staphylococcus aureus*. Thus, our pilot data demonstrated that the green synthesis of CM-AgNPs would be considered a good candidate for the treatment of HCT116 cancer cells, with its strong antioxidant activity and antibacterial effects.

## 1. Introduction

In recent decades, the nanotechnology is considered as one of the most promising research areas that has been an emerging branch in multidisciplinary fields [1,2]. Due to its excellent biological activities, the researchers are very interested in discovering many metallic nanoparticles, including the silver nanoparticles. The silver nanoparticles (AgNPs), whose sizes range between 1 nm and 100 nm, are mostly used in cosmetics, therapeutics, environmental and food packing, as well as for their antibacterial, anticancer, and antimicrobial properties in various industries, research into their anticancer activities [3,4,5,6]. Various synthetic methos such as physical, chemical, and biological are used for the synthesis of AgNPs [7]. Among these methods, the biological method is considered one of the most favorable for the synthesis of AgNPs due to its less toxicity and uniform particle size [8,9].

Currently, research shows that AgNPs have strong antibacterial and antifungal effects (which are very helpful in dental materials), wound healing effect, and they regenerate the damaged cells and remove saprophytic muscle [10,11,12,13,14,15]. Additionally, several reports have also mentioned that the AgNPs take part in modulating cytokines in wound repairs [14,16]. The antibacterial properties of AgNPs have been found to have great potential in the dental materials [17]. Additionally, cancer is considered one of the world’s most devastating diseases due to its high mortality rate and lack of effective treatment. All over the world, researchers are trying to find a leading metallic nanoparticle to overcome these problems. Thus, in comparison to the traditional treatment, AgNPs-based drugs in cancer therapy show great advantages with low treatment cost, low side effects, antibiotic resistance, and target orientation [18,19]. The occurrence of carcinogenesis is caused by free radicals which damage the DNA and chromosomal aberrations, and modify gene expression, which may be responsible for different cancers, such as colon cancer HT29, breast cancer MCF-7, liver cancer HepG2, cervical cancer HeLa, and lung cancer A549 in vitro [20,21,22,23,24]. In addition, AgNPs can induce cancer cells to produce a large number of reactive oxygen species and reduce the level of glutathione [25,26,27]. Hence, cancer nanomedicines have gained the promising interests of cancer therapies as a novel, efficient, and attractive strategy.

Chuanmingshen (CM) is widely distributed in most areas of China. CM processes the pharmacological effects of strengthening the brain, reducing lung heat, strengthening the spleen and liver, and reducing body fat [28]. Therefore, this study reports that CM contains a great number of natural ingredients. For instance, it has been demonstrated that ferulic acid, carotene, and neolilac chromatosterone were identified from the alcohol extract of CM [29]. Based on this, our research team chose CM as the research subject to obtain CM-AgNPs for its biological effects.

The biosynthesized CM-AgNPs were generally characterized by UV-Vis spectroscopy, FE-TEM, SEM, EDX spectroscopy, DLS, and FTIR spectroscopy [30,31]. Furthermore, this is the first report of biosynthesis of CM-AgNPs from the root of Chuanmingshen as a substrate used for the treatment of cancer cells in vitro analysis. The CM-AgNPs were assessed for their antioxidant activity against DPPH free radicals. Furthermore, the antibacterial activity of CM-AgNPs was investigated. Cytotoxicity of CM-AgNPs on four cancer cell types (MKN45, HCT116, A549, and HepG2 cells) and human lung fibroblasts cells (MRC-5) were examined in vitro. Furthermore, the effects of CM-AgNPs on HCT116 cells were emphatically analyzed by Hoechst staining, clone formation, Annexin V-FITC, and Mitochondrial Membrane Potential (MMP) assay. Ultimately, we intend to further investigate the molecular mechanism of HCT116 cell apoptosis induced by CM-AgNPs with reactive oxygen species (ROS) assay and western blot to determine their biological applications in cancer therapy.

## 2. Results

### 2.1. High-Performance Liquid Chromatography-Mass Spectrometry (HPLC-MS) Analysis of Chuanminshen Extract

**Figure 1:**
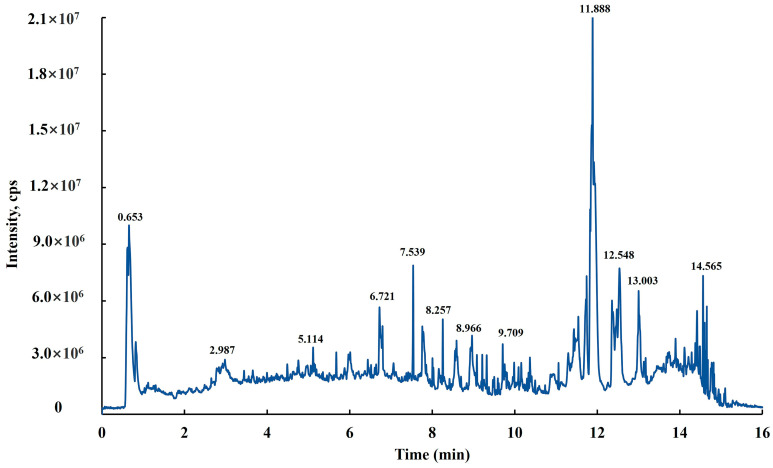
LC-MS spectra of *Chuanminshen* extract.

Through the analysis of the profiles detected in cation mode, 65 peaks were determined to be bioactive chemicals in *Chuanminshen* extract by LC-MS chromatography (Figure 1). The results implied that the six main chemicals contained in CM extract were ethers, isoflavans, polyphenol, diterpenoids, and amino acids. It also contained some other organic acids and derivatives, nucleosides, nucleotides, and analogues, and phenylpropanoids and polyketides as small molecule components. It can be seen that the main components in the extract of *Chuanminshen* were phenols, flavonoids, and terpenoids, which was in accordance with the previous studies and played the key role in the biosynthesis of nanoparticles [32].

### 2.2. Optimization Condition Analysis of CM-AgNPs

**Figure 2:**
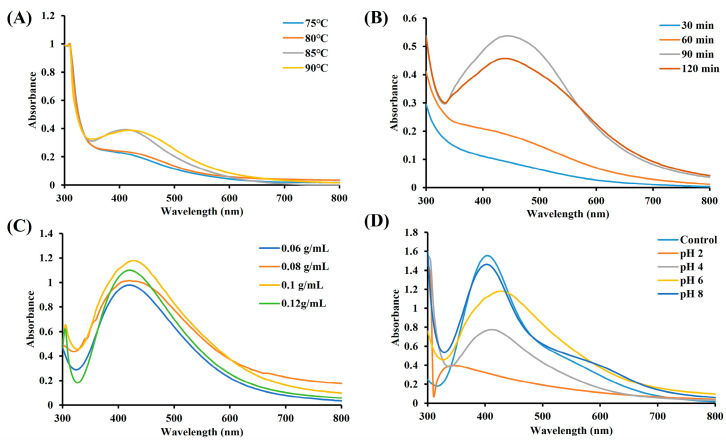
UV-Vis absorption spectra of CM-AgNPs showing the effects of temperature (**A**); time (**B**); varying plant extract concentration (**C**); and pH (**D**).

Figure 2A shows that the intensity of the absorption peak changed with the increase of temperature. The absorption peak increased significantly when the temperature reached 85 °C. Thus, the results show that the increase of temperature was conducive to the synthesis of CM-AgNPs. Additionally, the increase in time also had a positive effect on the synthesis of CM-AgNPs. Therefore, the maximum absorption peak appeared when the reaction time reached 90 min. Hence, 90 min was chosen as one of the optimal conditions for the synthesis of CM-AgNPs (Figure 2B).

As shown in Figure 2C, the absorption peak intensity increased with the increase of aqueous extract of CM concentration. The absorption peak intensity reached itsmaximum value when the concentration was 0.1 g/mL. Furthermore, the maximum absorption wavelength of the synthesized nanoparticles gradually appeared with the condition of pH 6. This meant that the environment (pH 6) contributed to the large formation of CM-AgNPs (Figure 2D).

### 2.3. Characterization of CM-AgNPs

**Figure 3:**
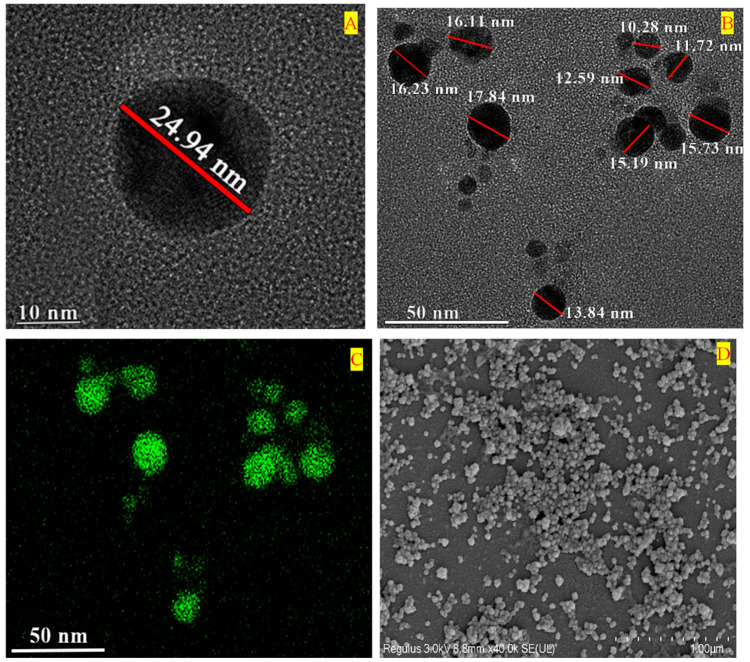

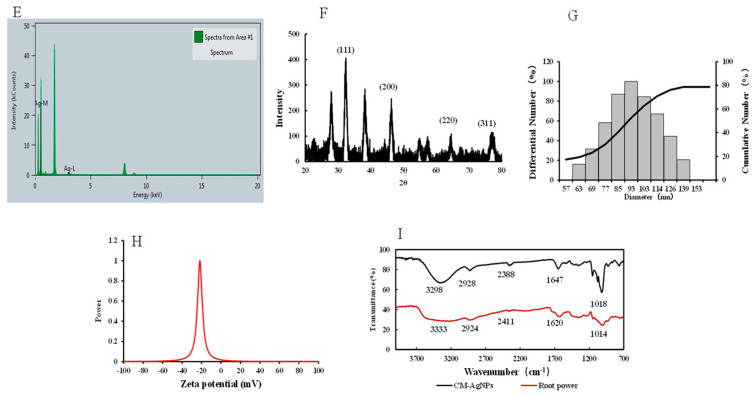
(**A**,**B**) FE-TEM images of spherical CM-AgNPs; (**C**) Elemental mapping distribution of CM-AgNPs; (**D**) SEM images of CM-AgNPs; (**E**) EDX spectra of CM-AgNPs; (**F**) XRD spectra of CM-AgNPs; (**G**) Particle size distributions of CM-AgNPs with respect to intensity; (**H**) Zeta potential values of CM-AgNPs; (**I**) FTIR spectra of CM-AgNPs and CM root power.

The FE-TEM results showed that the biosynthesized CM-AgNPs were spherical in shape, with sizes ranging from 10 to 25 nm, respectively (Figure 3A,B). In addition, the elemental mapping results showed the distribution of silver in the isolated nanoparticles, as shown in Figure 3C. The shape and surface morphology of the CM-AgNPs were examined by SEM analysis. The CM-AgNPs were spherical in morphology and evenly distributed (Figure 3D), and the purity of CM-AgNPs was analyzed using EDX. The EDX spectra demonstrated that the highest optical absorbance peak was at 2.3 and 3 keV, respectively, which correspond to the characteristic peaks of silver nanoparticles(Figure 3E). There has been a characteristic diffraction spectrum in every phase. The four diffraction peaks in the whole spectrum of 2θ value of silver nanoparticles were measured at 38.12°, 44.28°, 64.23°, and 77.47°, assigned to the peaks of (111), (200), (220), and (311) lattice planes of Bragg’s reflection, as shown in Figure 3F. As a result, the Ag element was found in CM-AgNPs. The hydrodynamic size distribution profiles of CM-AgNPs were obtained by DLS analysis for intensity (Figure 3G). The Z-average hydrodynamic diameter of nanoparticles was 89.56 ± 0.64 nm with a PDI of 0.262. According to the results of PDI value, the biosynthesized CM-AgNPs were moderately polydisperse. Zeta potential distribution of CM-AgNPs is shown in Figure 3H. The result indicates that the overall surface of CM-AgNPs carried negative charge, with an average value of −22.08 ± 2.12 mV.

The FTIR spectra of CM-AgNPs were compared against a spectrum of CM powder (positive control). FTIR results revealed that the root power showed the peaks at 3333 cm^−1^, 2924 cm^−1^, 2411 cm^−1^, 1620 cm^−1^, and 1014 cm^−1^ (Figure 3G). The strongest peak at 3333 cm^−1^ corresponded to the stretching of O-H bands of alcohol groups and flavonoids. The weak peak at 2311~2924 cm^−1^ was attributed to the C-H stretching of the methyl group. The medium peaks at 1620 cm^−1^ and 1014 cm^−1^ corresponded to the C=C bond and C-O bond, which were due to phenolic compounds and esters, respectively. Similar characteristic peaks were also found in the FTIR spectrum of CM-AgNPs, with the absence of a peak at 3298 cm^−1^. The results suggest that the major components in the aqueous extract might be responsible for forming the protective capping layers on the surface of CM-AgNPs, which acted as a dispersion and protection for the nanoparticles.

### 2.4. Stability Analysis

**Figure 4:**
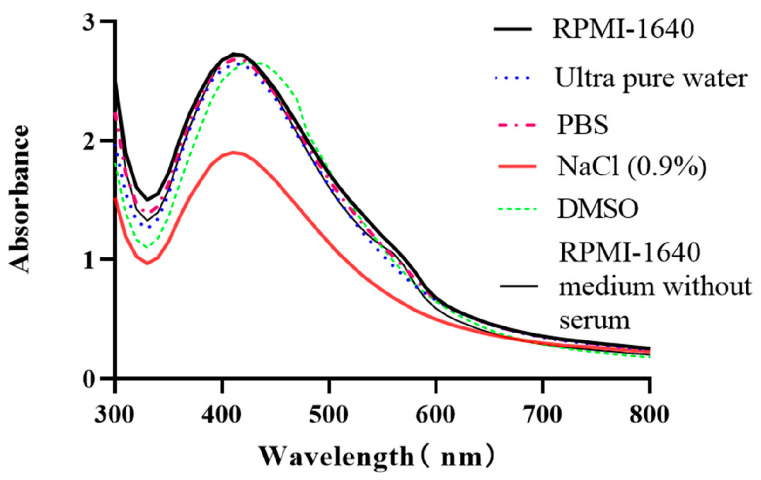
UV-Vis absorption spectra of CM-AgNPs in different media.

The stability of CM-AgNPs in different media were measured by a UV-Vis spectrophotometer. As illustrated in Figure 4, the UV-Vis characteristic absorption peak of the CM-AgNPs decreased significantly in the sodium chloride solution, which indicated that the stability of the CM-AgNPs in the sodium chloride solution was inferior. It was speculated that it might have been due to the electrostatic adsorption of the metal cations (Na^+^) in the system with the negatively charged substances attached to the surface of CM-AgNPs, resulting in different degrees of aggregation. In the dimethyl sulfoxide (DMSO) solution, the characteristic absorption peaks of the nanoparticles shifted, which indicate that the nanoparticles in the DMSO solution were quite instable. On the other hand, there was no significant change in the UV-Vis characteristic absorption peaks of CM-AgNPs in four solutions: RPMI-1640+10% fetal bovine serum (FBS), ultrapure water, phosphate-buffered saline (PBS), and RPMI-1640+1% FBS. CM-AgNPs were tested with favorable stability under the above conditions, which could meet the subsequent in vitro anticancer activity test.

### 2.5. Antioxidant Activity of CM-AgNPs

**Figure 5:**
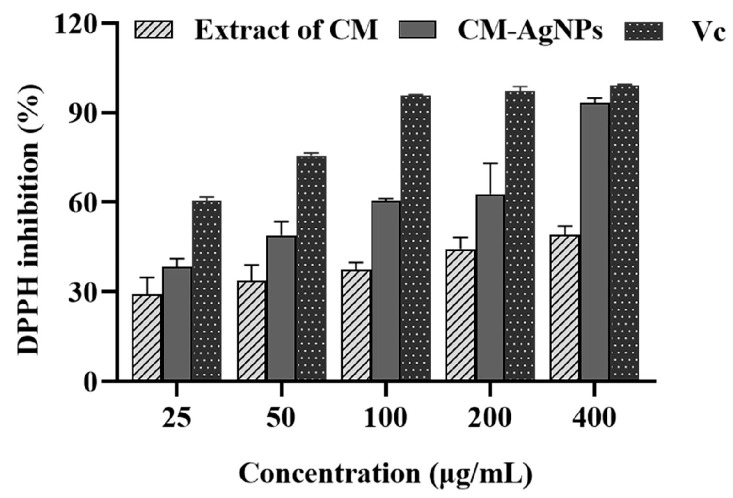
DPPH radical scavenging activity analysis of CM-AgNPs. Note: The DPPH radical scavenging effect of CM-AgNPs (25, 50, 100, 200, 400 μg/mL) was detected by DPPH radical scavenging assay. *Chuanminshen* extract (25, 50, 100, 200, 400 μg/mL) was used as negative control. Vc (25, 50, 100, 200, 400 μg/mL) was used as standard control. The data were expressed as mean ± standard error (*n* = 3).

The antioxidant effect of the generated CM-AgNPs was evaluated by the DPPH scavenging method. As shown in Figure 5, the scavenging rate of CM-AgNPs against DPPH radicals displayed the dose-dependent scavenging activity of the CM-AgNPs at 25–400 μg/mL. When the concentration of CM-AgNPs reached 100 μg/mL, the DPPH free radical scavenging rate was more than 50%. At this point the IC_50_ value was calculated to be 54.21 ± 4.41% μg/mL, which indicates that CM-AgNPs had a particular antioxidant activity and presented an apparent dose–effect relationship within a certain concentration range.

### 2.6. Antimicrobial Activity of CM-AgNPs

**Figure 6:**
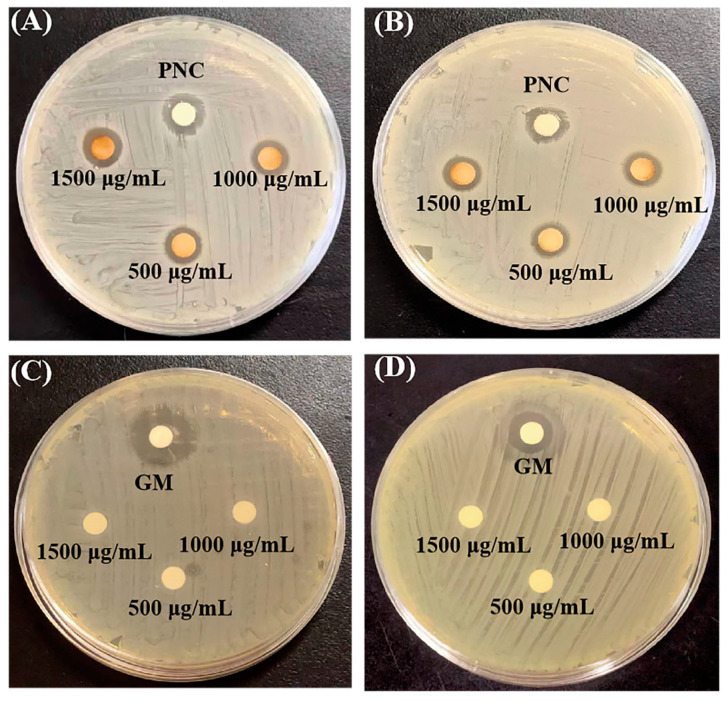
Zones of inhibition of CM-AgNPs and PNC against *E. coli* (**A**) and S. aureus (**B**); zones of inhibition of GM and the aqueous extract of Chuanmingshen against *E. coli* (**C**) and *S. aureus* (**D**).

Tables S1 and S2 display the inhibition zone values of CM-AgNPs against *E. coli* and *S. aureus*, comparing them with the aqueous extract of CM, Penicillin (PNC), and Gentamicin (GM). The results demonstrat that CM-AgNPs showed outstanding bacteriostatic effect against *E. coli*, and their bacteriostatic circle was 9.00 ± 0.90 mm, 12.57 ± 0.15 mm, and 16.63 ± 0.57 mm, respectively. In addition, CM-AgNPs also show excellent antibacterial effects against *S. aureus*, and the antibacterial circle was 9.10 ± 0.67 mm, 11.9 ± 0.21 mm, and 16.3 ± 0.56 mm, respectively. The results showed that the antibacterial activity (zone of inhibition) of CM-AgNPs significantly increased compared with the aqueous extract and positive drugs (PNC) at concentration of 500–1500 μg/mL (Figure 6A,B). On the other hand, the aqueous extract of CM in the determination of mass concentration range (500–1500 μg/mL) showed no bacteriostatic effect (Figure 6C,D).

**Figure 7:**
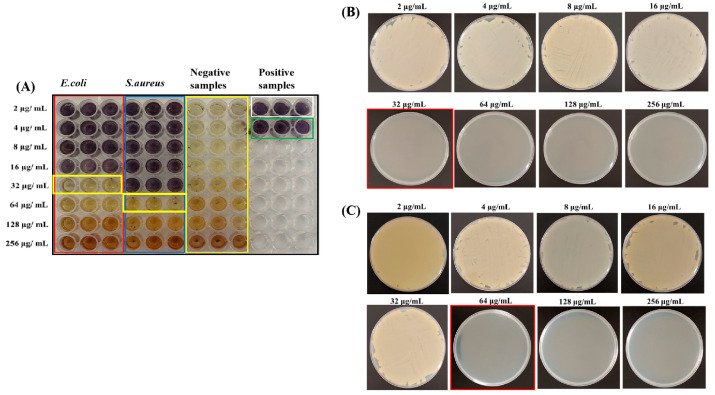
Analysis of MIC (**A**) and MBC (**B**,**C**) results against *E. coli* and *S. aureus* by CM-AgNPs.

The MIC and MBC of CM-AgNPs against *E. coli* and *S. aureus* were 32 μg/mL and 64 μg/mL, respectively (Figure 7A–C). It was found that CM-AgNPs (500–1500 μg/mL) displayed obvious inhibitory effects on the two kinds of tested bacteria with concentration dependent manner.

### 2.7. In Vitro Inhibitory Proliferative and Inducing Apoptosis Activity of CM-AgNPs in HCT116

#### 2.7.1. Cytotoxicity Evaluation of CM-AgNPs in Various Cells

**Figure 8:**
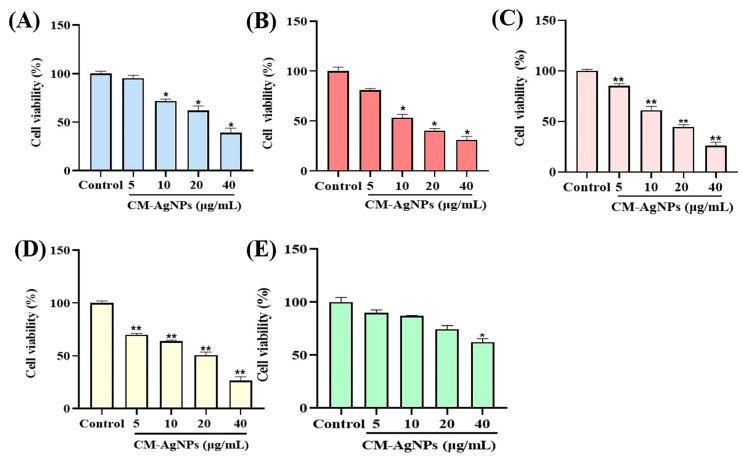
The results of proliferation of CM-AgNPs on MKN45 (**A**), HCT116 (**B**), A549 (**C**), HepG2 (**D**), and MRC-5 (**E**) cells. Note: MKN45 (**A**), HCT116 (**B**), A549 (**C**), HepG2 (**D**), and MRC-5 (**E**) were incubated with CM-AgNPs for 24 h. Cell viability was detected with CCK-8 reagent. The data were expressed as mean ± standard error (*n* = 3), compared to control (* *p* < 0.05; ** *p* < 0.01).

In vitro inhibitory proliferative activity of CM-AgNPs against MKN45, HCT116, A549, HepG2, and MRC-5 cells, using CCK8 assay, were shown in Figure 8A–E. CM-AgNPs displayed significant inhibitory effects on four types of tumor cells in a dose-dependent manner. However, there was no apparent cytotoxicity to MRC-5 cells and the IC_50_ value was calculated, as shown in Table S3. Consequently, the IC_50_ values of the MKN45, HCT116, A549, and HepG2 cells were 28.00 ± 3.33 μg/mL, 14.85 ± 1.01 μg/mL, 16.55 ± 0.27 μg/mL, and 16.51 ± 0.41 μg/mL, respectively. These results demonstrate that biosynthesized CM-AgNPs show a good cytotoxic effect on HCT116 cells. Therefore, HCT116 cells were chosen for further research.

#### 2.7.2. Clone Formation Assay Analysis

**Figure 9:**
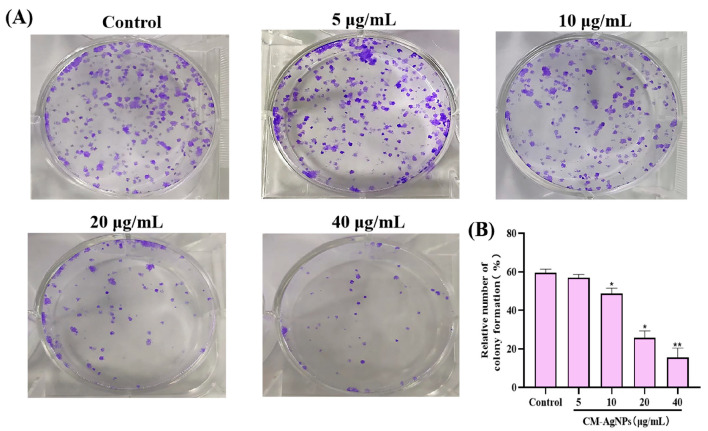
Colony formation assay of HCT116 cells treated by CM-AgNPs. Note: (**A**) was the cell colony formed after incubation of CM-AgNPs (0, 5, 10, 20, 40 μg/mL) with HCT116 cells. (**B**) was the cell colony formation rate. The data are expressed as mean ± standard error (*n* = 3), compared to control (* *p* < 0.05, ** *p* < 0.01).

To measure the proliferation ability of cultured cells, the clone forming efficiency was calculated. The results show that CM-AgNPs significantly reduce the clone ability of HCT116 cells at 20 and 40 μg/mL (*p* < 0.05, compared with control). As shown in Figure 9A, a significant difference in the number of cell clones between the CM-AgNPs-treated groups and control groups was observed, and the clone formation rate of cells in the normal control group was 59.3 ± 1.63%, while the clone formation rates of cells in the CM-AgNPs treated groups were 56.93 ± 1.40%, 46.0 ± 2.88%, 15.86 ± 2.76%, and 8.86 ± 1.61%, respectively (Figure 9B). The above results suggest that CM-AgNPs inhibited tumor growth by affecting the ability of HCT116 cells.

#### 2.7.3. Hoechst Stain Assay Analysis

**Figure 10:**
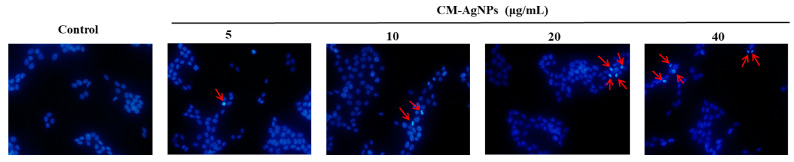
The morphological changes in the nuclei of HCT116 cells treated with various concentrations of (0, 5, 10, 20, 40 μg/mL) CM-AgNPs for 24 h were observed, with typical morphological changes of apoptotic nuclei. The photographs were taken under a fluorescence microscope (200×). The arrows indicate the apoptotic cells.

The morphology of the HCT116 cells were tested in the concentration range of 5–40 μg/mL for 24 h. The preliminary results indicate that nuclear fragmentation and shrinkage of chromatin occurred in the cells after treating HCT116 cells with CM-AgNPs for 24 h (Figure 10). These changes were more pronounced with increasing concentrations of CM-AgNPs.

#### 2.7.4. Apoptosis Analysis

**Figure 11:**
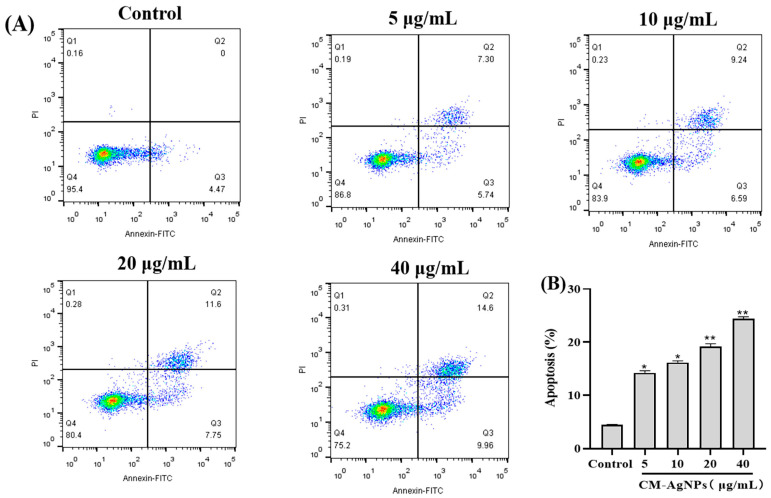
The apoptosis of HCT116 cells treated with CM-AgNPs for 24 h by Annexin V and PI staining. (**A**) is the apoptosis rate of HCT116 cells treated with CM-AgNPs (5, 10, 20, 40 μg/mL) for 24 h. The lower right quadrant is early apoptosis; the upper right quadrant is late apoptosis. (**B**) is the histogram of apoptosis rate ratio of HCT116 cells treated with CM-AgNPs for 24 h, where the total apoptosis rate is the sum of the early and late apoptosis rates. The data are expressed as mean ± standard error (*n* = 3) compared to control (* *p* < 0.05, ** *p* < 0.01).

The analysis showed that the apoptotic rate (14.22 ± 0.40%, 16.17 ± 0.32%, 19.19 ± 0.52% and 24.35 ± 0.57%) of HCT116 cells gradually increased with the increase of the CM-AgNPs (5–40 μg/mL) concentration, compared to the control group (Figure 11A). Among them, HCT116 cells showed an increase in late apoptosis. The statistical results of the apoptosis rate are shown in Figure 11B.

#### 2.7.5. ROS Production Level Analysis

**Figure 12:**
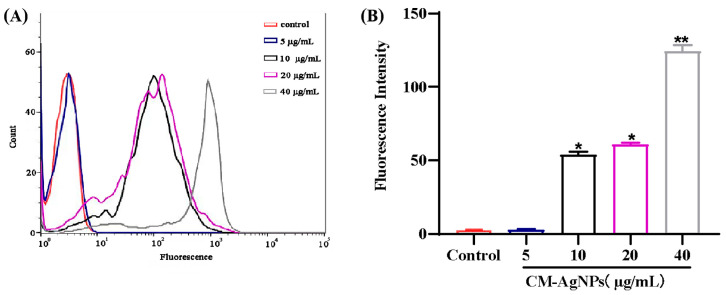
(**A**,**B**). Detection of ROS generation detected in HCT116 cells by DCFH-DA probe. Figure 11A shows the ROS production after the HCT116 cells treated with CM-AgNPs (5, 10, 20, 40 μg/mL for 24 h; Figure 11B shows the change of DCF fluorescence intensity generated by ROS in HCT116 cells treated with CM-AgNPs (5, 10, 20, 40 μg/mL). The data are expressed as mean ± standard error (*n* = 3), compared with positive control group (* *p* < 0.05, ** *p* < 0.01).

As shown in Figure 12A,B, the ROS production was proportional to the increase of concentration (10–40 μg/mL) of CM-AgNPs. The ROS production was almost 0 at 5 μg/mL CM-AgNPs, whereas the level of ROS production was maximum at 40 μg/mL, as shown in Figure 12B. Thus, the results of ROS production were compared with positive control (*p* < 0.01 or *p* < 0.05), and the killing effect of CM-AgNPs on HCT116 cells were recorded, which was closely related to the production of large amounts of ROS.

#### 2.7.6. MMP Analysis

**Figure 13:**
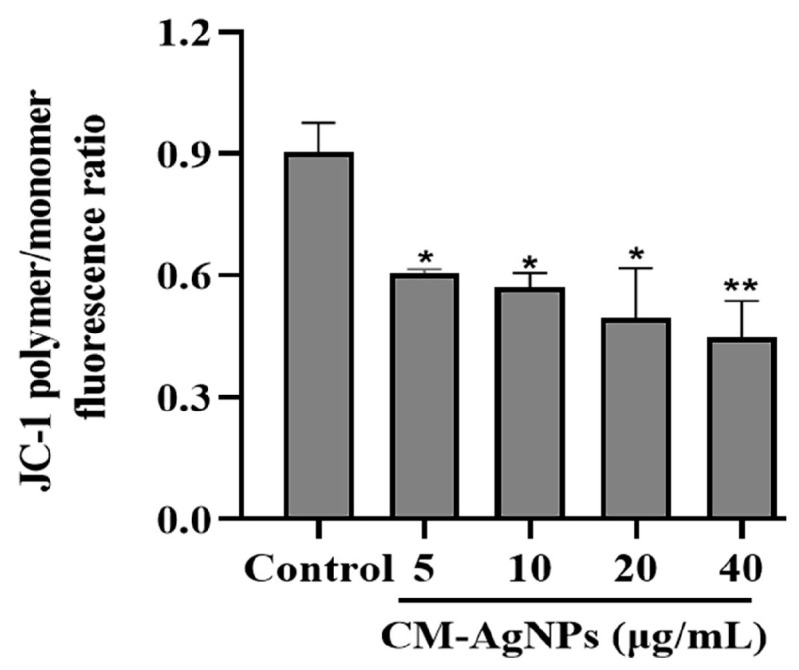
The detection of changes in mitochondrial membrane potential of HCT116 cells after 24 h of CM-AgNPs treatment was performed by JC-1 staining. A relative proportion of polymer and monomer fluorescence was commonly used to measure the mitochondrial depolarization ratio. The data are expressed as mean ± standard error (*n* = 3), compared with the control (* *p* < 0.05; ** *p* < 0.01).

As shown in Figure 13, the change of MMP in the cells were observed under the stimulation of CM-AgNPs. Compared with the control group, the MMP in the cells treated with CM-AgNPs at 5–40 μg/mL was markedly decreased (*p* < 0.01 or *p* < 0.05), whichmeant that HCT116 cells stimulated with CM-AgNPs could lead to a decrease in MMP analysis.

#### 2.7.7. Western Blot Analysis

**Figure 14:**
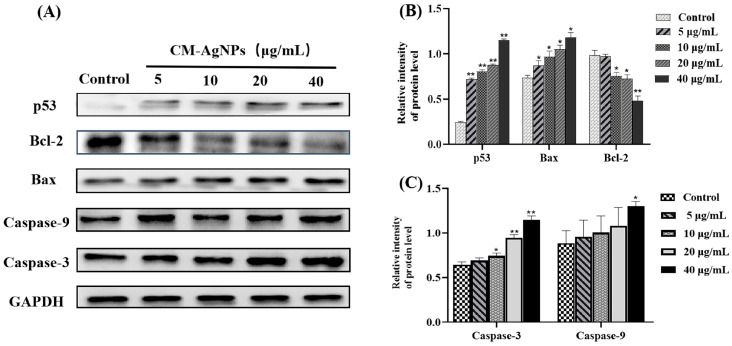
(**A**–**C**). The effect of CM-AgNPs on the expression of apoptosis-related proteins in HCT116 cells, detected by Western Blot. Note: (**A**) HCT116 cells p53, Bax, Bcl-2, Caspase 3 and Caspase-9—protein expression changes; (**B**) p53 HCT116 cells, Bax, Bcl-2 protein grey value analysis; (**C**) HCT116 cells Caspase 3, Caspase-9 protein grey value analysis. The data are represented as the average ± standard error (*n* = 3), compared with the control group (Control) (* *p* < 0.05, ** *p* < 0.01).

**Figure 15:**
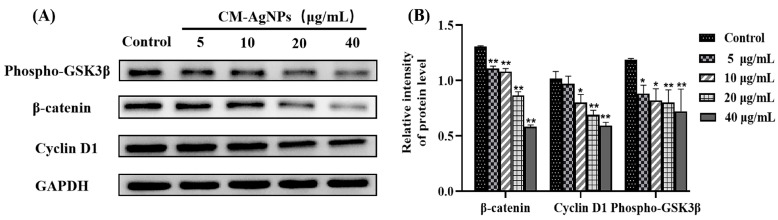
(**A**,**B**). The effect of CM-AgNPs on the expression of phospho-GSK-3β, β-catenin, and Cyclin D1 proteins in HCT116 cells, detected by Western Blot. Note: (**A**) the changes in the expression of phospho-GSK-3β, β-catenin, and Cyclin D1 in HCT116 cells; (**B**) protein grayscale analysis of phospho-GSK-3β, β-catenin, and Cyclin D1 in HCT116 cells. The data represent an average standard error (n = 3) compared with the control group (Control) (* *p* < 0.05, ** *p* < 0.01).

Figure 14 indicates that the expression of p53, Bax, and Caspase-3 proteins in HCT116 cells was increased (*p* < 0.01 or *p* < 0.05, compared with the positive control). Nevertheless, the Bcl-2 expression gradually decreased with the increase of the CM-AgNPs concentration (*p* < 0.01 or *p* < 0.05). These results revealed that CM-AgNPs (5–40 μg/mL) could increase the expression standards of p53, Bax, Caspase-3, and Caspase-9 proteins, and downregulate the expression levels of Bcl-2 proteins which induce the apoptosis. As shown in Figure 15, the expression of β-catenin, phosphor-GSK3β, and Cyclin D1 in the HCT116 cells decreased as a dose dependent (*p* < 0.05, *p* < 0.01).

## 3. Discussion

To a certain extent, the chemically synthesized AgNPs are not conducive to their biological applications [33]. The CM-AgNPs synthesized from CM extracts were non-toxic, and the CM extract had the advantages of being easily sourced and easily available. Most importantly, the reaction conditions were mild, safe, and environment-friendly [34,35]. The color change to dark yellow-brown from the original pale yellow has been implicated to confirm the formation of CM-AgNPs [36,37]. The chemical composition of CM-AgNPs is a key factor in determining its performance and application. The composition and structure of CM-AgNPs were analyzed by TEM, SEM, XRD, EDS, FTIR, and DLS. Combined with the characterization of CM-AgNPs, they were approximately spherical and small. Small-sized AgNPs possess prominent activity and permeability, which are widely used in the field of biomedicine. As mentioned in the literature review, the biologically synthesized AgNPs by plant extract exhibited antibacterial activity against *E. coli* and *S. aureus* [38,39]. The same results were obtained for CM-AgNPs. Additionally, as described in previous studies, the small size of AgNPs allows them to easily pass through the cytomembrane and cytoderm, which change the permeability of the plasma membrane and affect the cell homeostasis of microorganisms, inducing the chain of events that cause bacterial death [40,41].

Furthermore, we found that the CM-AgNPs exhibited marked antioxidant activity. The CM-AgNPs showed scavenging activity substantially better than the plant extract. This result was consistent with Arif M et al. [42], who summarized that the antioxidant properties of AgNPs were attributed to the phytochemicals, flavonoids, terpenoids, glycosides, and alkaloids with Ag^+^, which could act as antioxidants and reduce agents by single electron and hydrogen atom transfer [43,44,45]. Studies also confirmed that naturally occurring substances were renowned sources of specific biomolecules with antioxidant potentials [46]. Similarly, there were many effective bioactive components in the extract of CM. These substances attached to the surface of CM-AgNPs and gave them antioxidant activity.

In recent years, the antitumor activities of AgNPs against cancer cells have been widely observed. AgNPs synthesized by *Melissa officinalis* and *Lantana camara* leaf extract displayed remarkable antitumor activities against MCF-7 and A549 cell lines [47,48]. Usually, increased doses will cause more and more tumor cell apoptosis. It has been investigated by Rohini Pungle et al. [Year]. The AgNPs prepared by *Artemisia turcomanica* leaf extract indicated anticancer effects on the A549 human non-small-lung cancer cell lines with both dose dependents [49]. At the same time, it has been reported that AgNPs obtained from plant extract inhibited the growth of MCF-7 cells [50,51]. Based on the above description, the reproduction and growth of the tumor cells can be suppressed by AgNPs. We have successfully verified the cytotoxicity of CM-AgNPs in vitro. Our results demonstrate that CM-AgNPs exerts outstanding cytotoxic effects on HCT116 cells with IC50 of 13.75 ± 2.07 μg/mL. The CM-AgNPs (5–40 μg/mL) had significant effects on cell morphology and could significantly induce cell apoptosis. The underlying mechanism of CM-AgNPs for HCT116 cells apoptosis was the enhanced generation of ROS. Cellular stress response caused by reactive oxygen species accumulation is an important triggering mode of cell apoptosis induced by physicochemical factors. In general, ROS is controlled at regular levels by normal cellular antioxidant defense mechanisms, and the normal physiological activities of cells are not affected. However, large amounts of ROS can cause oxidative stress, which reduces the activity of biomacromolecules and damages subcellular organelles [52]. In this research, the results showed that elevated ROS levels in HCT116 cells treated with CM-AgNPs triggered oxidative stress. A large amount of ROS could cause serious damage to the structure and function of cells. There was one further point to make; another mechanism by which CM-AgNPs induce apoptosis in HCT116 cells may be feasible to involve endogenous mitochondrial pathways. Endogenous mitochondrial pathways were regulated by pro-apoptotic and anti-apoptotic proteins of the Bcl-2 protein family [53]. The Bcl-2 family affected the permeability of the mitochondrial outer membrane and inner membrane, and it was the main regulator of mitochondrial apoptotic pathway. Bax protein was the main mediator of the mitochondrial pathway [54,55]. After activation of Bax, its expression was upregulated. The permeability of mitochondrial membrane was damaged. Bcl-2 was a negative modulator of cell death. It could have been due to the protection of many types of cells from apoptosis when exposed to external stimuli. Apoptosis can be regulated by inhibiting Bcl-2 activity. As mentioned above, Bcl-2 family proteins can jointly determine whether cells enter the apoptotic process through the mitochondrial pathway. Caspase-9 belongs to cysteine protease, which is closely related to the pro-apoptotic signal [56]. Activation of caspase-9 will cleave and activate the downstream effecting group Caspase-3. The effector group Caspase-3 induces cell apoptosis by cutting the site of specific aspartic acid residues in the cell protein [57]. Our studies demonstrated that CM-AgNPs had certain toxic effects on HCT116 cells and could inhibit the proliferation of HCT116 cells. In view of this, we further investigated the apoptosis of HCT116 cells induced by CM-AgNPs using Hoechst staining and Annexin V-FITC assay. Hoechst staining analysis confirmed that CM-AgNPs could shrink HCT116 cells. Meanwhile, it had been proven that HCT116 cells underwent apoptosis after 24 h treatment with CM-AgNPs by Annexin V-FITC analysis. It was mainly concentrated in the early stage and it significantly increased compared with the control group. The above results further revealed that CM-AgNPs significantly promoted the apoptosis of HCT116 cells.

Furthermore, the short-term changes of mitochondrial membrane potential induced by CM-AgNPs were investigated using a JC-1 probe. The red/green ratio of HCT116 cells was decreased after 24 h treatment with CM-AgNPs (5–40 μg/mL). To further confirm whether CM-AgNPs-induced apoptosis of HCT116 cells through the mitochondrial pathway, we analyzed the protein expressions of p53, Bax, Caspase-3, and Bcl-2 by Western blot. The results represented that CM-AgNPs could significantly increase the expressions of proapoptotic protein p53, Bax, Caspase-3, and Caspase-9 in HCT116 cells (*p* < 0.01 or *p* < 0.05). Simultaneously, CM-AgNPs could significantly reduce the expression level of anti-apoptotic protein Bcl-2 (*p* < 0.01 or *p* < 0.05). Overall, CM-AgNPs regulated the expression of mitochondrial pathway-related apoptotic protein to promote the apoptosis of HCT116 cells. In addition, the influence of AgNPs protein targets and the signaling pathway also vary in different tumor cells [58,59]. Wnt/β-catenin pathway of abnormal activation is closely related to the occurrence of the digestive tract tumor development [60]. β-catenin is the key signal on the channel proteins, which can affect the p-GSK3β and Cyclin D1 expressions. The results showed that the CM-AgNPs inhibited cell proliferation by Wnt/β-catenin pathway activation. The results provide an effective reference for the application of AgNPs in cancer treatment and the use of nanotechnology in the biomedical field.

## 4. Materials and Methods

### 4.1. Preparation of CM Root Extract

CM roots were obtained from the Food Technology Laboratory, Yantai University, Yantai, Shandong Province of China. The roots of CM were washed and dried, then grinded to make powder. Five grams of powder was mixed with 50 mL distilled water and autoclaved for 30 min to obtain the aqueous root extract. After extraction, the extract was filtered using Whatman filter paper (No. 2, Shanghai Jinpan Biotechnology Co., LTD, Shanghai, China) and centrifuged at 11,000 rpm for 10 min to discard the unwanted sediments. After centrifugation, the supernatant was collected, and the final volume was made at 100 mL by adding sterile DDW (double distilled water) and then was stored at 90 °C for further study.

### 4.2. High-Performance Liquid Chromatography-Mass Spectrometry (HPLC-MS) Analysis

For the compositional analysis of the *Chuanminshen* extract, high-performance liquid chromatography-mass spectrometry (HPLC-MS) was used. The samples were separated using an Agilent 1290 Infinity LC Ultra High-Performance Liquid Chromatography system (UHPLC, Agilent, Santa Clara, CA, USA) with a HILIC column. The column temperature was 25 °C; the flow rate was 0.5 mL/min; and the injection volume was 2 μL. The LC-MS detection was performed in electrospray ionization (ESI) positive ion mode. The temperature of the ion source was set at 600 °C, the spray voltage (ISVF) ± 5500 V, the collision energy was 35 ± 15 eV, and 10 fragmentation profiles were collected in each scan. The retention time (in minutes), peak area, peak height and mass spectral pattern of the test samples were compared with the data in the database to determine the phytochemical constituents.

### 4.3. Synthesis of CM-AgNPs

AgNO_3_ solution (final concentration of 1 mM) was mixed with 100 mL of CM (pH 7.0) extract and then kept at 85 °C for 120 min to reduce ions to atoms. After incubation at 85 °C, the change of color of the reaction mixture indicated the formation of AgNPs (from light yellow to dark brown). To collect and purify the synthesized AgNPs, the reaction mixture was centrifuged at 15,000 rpm for 15 min and the pellets were washed with sterile distilled water. Finally, the purified nanoparticles were dried at room temperature and used for characterization and biological applications in vitro. The CM-AgNPs, before reaction, was used as control in all experiments.

### 4.4. Optimization of Preparation Conditions of CM-AgNPs

To optimize the reaction conditions of CM-AgNPs, the effect of various temperatures (75 °C, 80 °C, 85 °C, 90 °C), different pH values (pH 2, 4, 6, 7, 8), time (30, 60, 90, 120 min) and different concentration (0.06, 0.08, 0.10, 0.12 g/mL) of the extract concentration for the optimal formation of CM-AgNPs were tested and confirmed by UV–Visible spectrophotometer.

### 4.5. Characterization of CM-AgNPs

The synthesis of CM-AgNPs was confirmed using UV-Vis spectrophotometer (2100 Pro, Amersham Bioscience Crop, UK (Slough, Buckinghamshire, UK), while the size, morphology, purity, and elemental distribution and mapping of the CM-AgNPs were characterized using FE-TEM, EDX with a JEM-2100F (JEOL) electron microscope (JEOL Ltd., Tokyo, Japan) operated at 200 kV and SEM (JSM-7800F, JEOL, Japan). For morphological observations, a drop of purified CM-AgNPs solution was put on the carbon-coated copper grid and kept at 60 °C to evaporate the solvent, and then the morphology was observed using FE-TEM. Elemental mapping was measured for exhibiting the relative location of the target elements by EDX. Additionally, the morphology of CM-AgNPs was observed more directly using SEM.

XRD is a common means to determine the phase structure of nanomaterials. Generally, the phase composition of nanomaterials was identified based on the position and strength of the XRD diffraction pattern. Every phase possesses its characteristic diffraction spectrum. The Ag elements presented in the CM-AgNPs was determined by comparing the map of the measured samples with the peak position of Ag in the standard card. The CM-AgNPs were placed on the slide and tested with X-ray diffractometer (Rigaku SmartLab SE, Tokyo, Japan). The CM-AgNPs were scanned over the diffraction angle (2θ) ranging from 20 to 80°. The sweep speed was 2°/min. One light source was Cu-Kα ray, the tube voltage was 40 kV, and the current was 40 mA.

The hydrodynamic diameters and polydispersity index (PDI) of CM-AgNPs were analyzed using the DLS (DLS-Photal, Otsuka Electronics, Tokyo, Japan) technique at 25 °C. As a reference, the refractive index, viscosity, and dielectric constant of a dispersive medium of pure water were 1.3328, 0.8878, and 78.3, respectively. The CM-AgNPs were added to ultrapure water and sonicated for 30 min, and the particle size distribution was measured using a Malvern spray analyzer (Malvern Panalytical, Shanghai, China).

In the field of nanomaterials, the positive and negative surface charges of nanoparticles are mostly determined by Zeta potential, which can guide the modification or application of different systems for future studies. To find the charge on the AgNPs, 2 mL of the solution of CM-AgNPs was added into the colorimetric dish of the particle size sample tank. Whereafter, a colorimetric dish containing the sample was inserted into the sample tank and the Zeta potential was measured. A higher potential indicates that the nanosilver surface is more charged and will tend to repel each other, thus maintaining the stability of the whole system. And the dividing line for the dispersion stability of nanoparticles in the aqueous phase is generally considered to be at +30 mV or −30 mV, so it indicates that the aqueous phase dispersion system of CM-AgNPs is relatively stable.

FTIR spectroscopy was utilized to classify the nature of possible biomolecules responsible for protecting the capping layer and stabilizing the CM-AgNPs. FTIR analysis was operated on a PerkinElmer Spectrum One FTIR spectrometer (Thermo Fisher Scientific, Waltham, MA, USA) in the range of 700–4000 cm^−1^ at a resolution of 4 cm^−1^. The data of experimental AgNPs were compared with the Chuanmingshen root.

### 4.6. Stability Analysis of CM-AgNPs in Different Media

The stability of CM-AgNPs in different solutions was investigated by observing their characteristic absorption spectra via diverse media. The samples and test solutions were mixed evenly and placed at room temperature for 40 min. Three milliliters of the mixed solution was added to the quartz dish, and then measured by UV-Vis spectrophotometer at 300–800 nm.

### 4.7. Antibacterial Assay of CM-AgNPs

The bacteriostatic action of CM-AgNPs was determined by disc diffusion method, which aimed to explore the inhibitory effect of CM-AgNPs on *E. coli* and *S. aureus* on Muller-Hinton (MH) agar plates. Briefly, the *E. coli* and *S. aureus* bacteria were evenly cultured on the MH agar plates and incubated at 37 °C overnight. PNC and GM were used as a positive control. Meanwhile, the sterile disc paper was soaked with various concentration CM-AgNPs solution (500, 1000, and 1500 µg/mL) and put on the surface of cultured plates and incubator at 37 °C for further 24 h. After 24 h, the bacteriostatic circle diameter (or zone of inhibition) of CM-AgNPs and antibiotics were compared with each other.

### 4.8. The Minimum Inhibitory Concentration (MIC) and Minimum Bactericidal Concentration (MBC) Assay of CM-AgNPs

The MIC value of CM-AgNPs against *E. coli* and *S. aureus* were analyzed using 3-(4,5-dimethylthiazol-2-yl)-2,5-diphenyltetrazolium bromide (MTT) colorimetric assay as mentioned by Patel et al. [61]. Briefly, the bacterial suspensions were added into a 96-well plate with various concentration of CM-AgNPs solutions (2, 4, 8, 16, 32, 64, 128 and 256 μg/mL). *E. coli* and *S. aureus* without CM-AgNPs were cultured as positive control. After incubation for 24 h at 37 °C, 10 μL (containing 5 mg/mL MTT) solution was added to each well and incubated at 37 °C for 4 h in dark. The color change of the reacted solution was observed to determine the MIC value. The experimental group samples were evenly coated onto agar culture medium. The MBC was the absence of colony growth on solid medium.

### 4.9. In Vitro DPPH Radical Scavenging Assay

The oxidation resistance activity of CM-AgNPs against free DPPH radicals was determined by an improved DPPH scavenging method [62]. Ascorbic acid (Vitamin C) and DPPH were obtained from China National Pharmaceutical Group Co. LTD Beijing. In brief, the DPPH solution (0.1 mM) was mixed with different concentrations of CM-AgNPs solution (25–200 μg/mL), and then the solution was mixed well. The samples were kept in a dark place for 30 min at room temperature and the DPPH activity was determined at absorbance of 517 nm. The DPPH free radical scavenging rate was estimated by the following formula:

[(Absorbance of control − Absorbance of the sample)/Absorbance of control] × 100.(1)

### 4.10. In Vitro Inhibitory Proliferative and Inducing Apoptosis Activity of CM-AgNPs in HCT116

#### 4.10.1. Cytotoxicity Evaluation of CM-AgNPs in Various Cells

The anti-cancer activity of CM-AgNPs was investigated on MKN45, HCT116, A549, HepG2 and MRC-5 cancer cells. All the cells were cultured and incubated at 37 °C containing 4% CO_2_. After 24 h, the cells were collected at their maximum growth rate (counted under the microscope), and then diluted into 3 × 10^3^ cells/well with cell culture medium. The cell suspension was added into a sterile 96-well plate and cultured again for 24 h until the cells attached to the 96-well plate’s surface. After that, CM-AgNPs (5–40 μg/mL) were added into the cell culture box for another 24 h. The supernatant of each well was discarded and 10 μL CCK8 solutions were added and incubated for 1 h. After incubation, the samples absorbance was measured at 450 nm. The absorption value of the untreated control sample was chosen as 100% cell viability.

#### 4.10.2. Hoechst Staining Assay

To confirm the apoptosis activity of CM-AgNPs in HCT116 cells, the change of HCT116 cells morphology were observed by Hoechst staining. The coverslips were prepared and soaked in 70% ethanol solution for 5 min and washed with PBS buffer solution. Then, the HCT116 cells (1 × 10^5^ cells/well) were cultivated in six-well plates containing coverslips and incubated for 24 h at 37 °C with 4% CO_2_. After 24 h, the medium was removed with 1 mL of fresh medium containing CM-AgNPs (5–40 μg/mL) and further incubated for 24 h in the same incubator. After 24 h, the supernatant in each well was discarded and the cells were fixed with a fixing solution for certain time and then washed with PBS buffer and then the cells were stained for 10 min. Finally, the morphological changes of HCT116 cell were observed by fluorescence microscopy.

#### 4.10.3. Clone Formation Assay

The clone formation assay for evaluating the inhibitory effect of the CM-AgNPs on HCT116 cells was also conducted, as described previously. HCT116 cells were transferred to six-well plates at a density of 500 cells/well with CM-AgNPs at a concentration of 5–40 μg/mL suspended in RPMI-1640 medium (Gibco|Life Technologies, Grand Island, NY, USA). The plates were cultured and incubated at 37 °C with 5% CO_2_ for one week. A week later, the cells were immobilized with 4% paraformaldehyde for 15 min and then washed repeatedly with PBS. After cell fixation, the cells were washed twice with cold PBS with being soaked stained with crystal violet (Beyotime, China) for 25 min and then washed with distilled water. Finally, the cell colonies were collected by photographing, and the clone formation rate was analyzed by the image J software (https://imagej.net/downloads, 2 October 2024, National Institutes of Health, NIH).

#### 4.10.4. Apoptosis Measurement

Annexin V-FITC reagent (Beyotime, Shanghai, China) was conducted to evaluate the apoptosis rate of the HCT116 cells. To begin with, HCT116 cells (5 × 10^4^ cell/well) were plated and exposed to different concentrations (5, 10, 20, 40 μg/mL) of CM-AgNPs for 24 h at 37 °C in a humidified atmosphere containing 5% CO_2_ and 95% air. After apoptotic stimulation, the cells were collected and gently resuspended with PBS for washing, and then the cell precipitates were resuspended in Annexin V-FITC buffer (195 μL). Subsequently, Annexin V-FITC (5 μL) and Propidium Iodide staining solutions (10 μL) were added to the cell suspensions and incubated under dark conditions for 30 min. After 30 min, cell apoptosis rates were analyzed by flow cytometry.

#### 4.10.5. Detection of ROS Level

ROS level was detected using 2,7-dichlorodihydrofluorescein diacetate (DCFH-DA, Beyotime, Shanghai, China). Firstly, HCT116 cells were seeded in six-well plates at 5 × 10^4^ cells/well and incubated with the CM-AgNPs at concentrations of 5, 10, 20, and 40 μg/mL. After 24 h of incubation, the HCT116 cells were collected and incubated with 10 μM DCFH-DA for 20 min in CO_2_ condition. The HCT116 cells were washed by serum-free medium to adequately remove DCFH-DA and analyzed ROS level by flow cytometry.

#### 4.10.6. MMP Measurement

MMP assay kit with JC-1 (Beyotime, Shanghai, China) was used to measure the apoptosis of HCT116 cells after treatment with CM-AgNPs (5–40 μg/mL) for 24 h. Cells were stained with 1 mL of serum-free cell culture medium and JC-1 staining solution at 37 °C for 20 min. Then, the cells were treated with JC-1 staining buffer (1×), and the flow cytometry was performed to analyze the red and green fluorescence ratio.

#### 4.10.7. Western Blotting

The HCT116 cells were lysed with ice RIPA buffer containing protease inhibitors cocktails. The scraped cells were lysed again on ice for 5 min, and then the supernatants were collected. Each concentration of the proteins was measured using BCA Protein Concentration Quantitation Kit (Beyotime, Shanghai, China) at a wavelength of 562 nm. Equal amounts of protein (30 μg) were separated by 10% SDS-PAGE gel and transferred to the PVDF membrane at low temperature. The membrane was blocked with 5% bovine serum albumin at room temperature for two hours. Each membrane was incubated with the following antibodies: GAPDH Bax, Bcl-2, p53, Caspase-3, Caspase-9, CyclinD1, p-GSK3β, and β-catenin. After incubation, the membrane was quickly washed, and the corresponding secondary antibody was added and incubated for 1 h in the shaking incubator. Finally, the ECL chemiluminescent solution was evenly added dropwise on the membrane and imaged in a gel imager. The dilution ratio of all antibodies in the Western blot was 1:1000.

#### 4.10.8. Statistical Analysis

All assays were repeated three times in parallel, and the results were expressed as means ± standard deviations (SD). A one-way ANOVA test was utilized to evaluate the data values of the treated and untreated (control) groups. The values of *p* < 0.05 were considered significantly.

## 5. Conclusions

CM extracts played the role of a reducer and stabilizer for the directly convenient synthesis of CM-AgNPs without the addition of toxic chemical reagents. The CM-AgNPs were widely characterized by UV-Vis absorption spectroscopy, FE-TEM, SEM, elemental mapping, EDX, XRD, DLS, and FTIR spectroscopy. The results showed that biosynthesized CM-AgNPs in various medium solutions had always been a relatively stable state. Moreover, the CM-AgNPs exhibited good potentials as a new type of antioxidant. Similarly, CM-AgNPs also showed excellent antimicrobial activities against pathogenic *E. coli* and *S. aureus*. In vitro studies, CM-AgNPs could selectively inhibit the proliferation and destroy the MMP of HCT116 cells and trigger the apoptosis of HCT116 cells by regulating the production of ROS level and the expression of pro-apoptotic proteins (Bax, p53, Caspase-3, Caspase-9) and anti-apoptotic proteins (Bcl-2). Meanwhile, CM-AgNPs inhibited HCT116 cell proliferation by the Wnt/β-catenin pathway.

Thus, our pilot data demonstrated that the CM-AgNPs was a good finding for the treatment of cancer cells as well as for the antibacterial activity of two common human pathogens, *E. coli* and *S. aureus*. The synthesized CM-AgNPs from the root extracts of CM plant was an economical approach and a viable alternative to conventional methods. Further studies will be necessary to explore further applications in the fields of biomedical sciences to uncover possible therapeutic benefits and potential for treating various cancers.

## Data Availability

The datasets generated for this study are available on request to the corresponding author.

## Supplementary Materials

The following supporting information can be downloaded at: https://www.mdpi.com/article/10.3390/molecules29235682/s1. Table S1: Bacteriostatic value of CM-AgNPs, PNC, GM, and the aqueous extract of chuanmingshen against *E. coli* and *S. aureus*; Table S2: Bacteriostatic value of the aqueous extract of chuanmingshen and GM against *E. coli* and *S. aureus*; Table S3. The IC_50_ values of MKN45, HCT116, A549, and HepG2 cells treated with CM-AgNPs.
