# Supplementary material for: Green Synthesis of Silver Nanoparticles (CM-AgNPs) from the Root of Chuanminshen for Improving the Cytotoxicity Effect in Cancer Cells with Antibacterial and Antioxidant Activities
Source: Molecules. 2024 Nov 30;29(23):5682. doi: 10.3390/molecules29235682 (PMC11643779; doi:10.3390/molecules29235682)
Supplement: Supplementary file 1 [file molecules-29-05682-s001.zip › molecules-3076891-supplementary.pdf]

Table S1. Bacteriostatic value of CM-AgNPs and PNC against *E. coli* and *S. aureus.*

Pathogenic microorganisms	Zone of inhibition (mm)*			
	PNC	500 μg/mL	1000 μg/mL	1500 μg/mL
E. coli	11.97±0.21	9.00±0.90	12.57±0.15	16.63±0.57
S. aureus	9.63±0.47	9.10±0.67	11.9±0.21	16.3±0.56

*Mean diameter of three discs; 20 μL of solutions containing partially purified CM-AgNPs. Note:PNC is a positive control. In the table, 500-1500 μg/mL was different concentrations of CM-AgNPs. Data were expressed as mean ± standard error (n=3).

Table S2. Bacteriostatic value of the aqueous extract of Chuanmingshen and GM against *E. coli and S. aureus.*

Pathogenic microorganisms	Zone of inhibition (mm)*			
	GM	500 μg/mL	1000 μg/mL	1500 μg/mL
E. coli	21.77±0.34	-	-	-
S. aureus	18.77±0.81	-	-	-

*Mean diameter of three discs; 20 μL of solutions containing partially the aqueous extract of chuanmingshen. Note: GM is a positive control. In the table, 500-1500 μg/mL was different concentrations of the aqueous extract of chuanmingshen. "-" indicates no data. Data were expressed as mean ± standard error (n=3).

Table S3. The IC_{50} values of MKN45, HCT116, A549, and Hep G2 cells treated with CM-AgNPs

Cell type	IC_{50} (μg/mL)
MKN45	28.00±3.33
HCT116	14.85±1.01
A549	16.55±0.27
HepG2	16.51±0.41

Note: The IC50 values of MKN45 cells, HCT116 cells, A549 cells and HepG2 cells in the table were calculated by GraphPad Prism software, and the data were expressed as mean ± standard error (n=3).
